# Dynamic Object Tracking on Autonomous UAV System for Surveillance Applications

**DOI:** 10.3390/s21237888

**Published:** 2021-11-27

**Authors:** Li-Yu Lo, Chi Hao Yiu, Yu Tang, An-Shik Yang, Boyang Li, Chih-Yung Wen

**Affiliations:** 1 Department of Aeronautical and Aviation Engineering, The Hong Kong Polytechnic University, Kowloon, Hong Kong, China; liyu.lo@connect.polyu.hk (L.-Y.L.); chi-hao.yiu@connect.polyu.hk (C.H.Y.); bryant.tang@connect.polyu.hk (Y.T.); chihyung.wen@polyu.edu.hk (C.-Y.W.); 2 Department of Energy and Refrigerating Air-Conditioning Engineering, National Taipei University of Technology, Taipei 10608, Taiwan; asyang@ntut.edu.tw

**Keywords:** UAV, object detection, object tracking, deep learning, Kalman Filter, autonomous surveillance

## Abstract

The ever-burgeoning growth of autonomous unmanned aerial vehicles (UAVs) has demonstrated a promising platform for utilization in real-world applications. In particular, a UAV equipped with a vision system could be leveraged for surveillance applications. This paper proposes a learning-based UAV system for achieving autonomous surveillance, in which the UAV can be of assistance in autonomously detecting, tracking, and following a target object without human intervention. Specifically, we adopted the YOLOv4-Tiny algorithm for semantic object detection and then consolidated it with a 3D object pose estimation method and Kalman filter to enhance the perception performance. In addition, UAV path planning for a surveillance maneuver is integrated to complete the fully autonomous system. The perception module is assessed on a quadrotor UAV, while the whole system is validated through flight experiments. The experiment results verified the robustness, effectiveness, and reliability of the autonomous object tracking UAV system in performing surveillance tasks. The source code is released to the research community for future reference.

## 1. Introduction

Unmanned aerial vehicles (UAVs) have revealed their unprecedented potential for commercial, military, and civil-government utilization in a wide range of applications such as infrastructure inspection [1], aerial photography [2], logistics [3], and so forth. The employment of a UAV incorporated with vision techniques is exclusively advantageous for tasks that require distinct visualization and robust perception, for example, aerial surveillance operations.

Surveillance plays a vital role in maintaining safety and security as it detects and prevents emerging unusual events. Many important tasks, such as information collection, military reconnaissance, target tracking, and even traffic management, have a connection to surveillance technologies. Nonetheless, a conventional surveillance mission is conducted through manual practice to identify targets, which is time-consuming, labor-intensive, tedious, costly, and risky for operators if entering some impassable regions. Hence, the development of a UAV as a surveillance tool is gaining a tremendous amount of popularity to reduce human effort significantly. A UAV is capable of assisting the surveillance activities by its agile maneuverability to approach confined areas of low accessibility and its visual functionality to capture the remote scene in real-time. In addition, an autonomous UAV without manual teleoperation has been shown as a cost-effective solution in resource optimization to aid routine surveillance in different industries. On behalf of mankind, an autonomous UAV is extremely helpful for continuously monitoring the movement of distant target objects.

In the past few years, the UAV research community has endeavored to enhance the tracking performance of vision-based surveillance in different application scenarios. For instance, Chung et al. [4] implemented the standard but relatively old techniques based on background subtraction and frame differencing to detect objects from an aerial robot. However, these methods work poorly with a moving UAV that has high-frequency vibrations in the camera motion. Then, Fang et al. [5] showed that a UAV system with a kernel-based mean shift algorithm [6] could not robustly track the target object with changing size and moving speed. Using a color-based tracker with multi-part representation, Teuliere et al. [7] used a UAV to autonomously track and chase a moving target. However, many environmental factors could result in malfunctions in their tests, such as low-resolution imagery, noise corruption, variation of illumination, especially when the background and targets’ color are similar. Other object tracking techniques like the drone-based mobile surveillance system with mobility-aware dynamic computation offloading and pan-tilt-zoom (PTZ) camera from Kim et al. [8], and the approach offered by Zhang [9] overload computational costs on the UAV platform. Due to the limited onboard computing resources, their algorithms could not perform in real-time and onboard directly. Alternatively, object tracking systems with multiple sensor data fusion suggested by Carrillo et al. [10] and Cho and Lee [11], Liu et al. [12] are also expected to increase the payload and battery power consumption of a UAV significantly, and thus contradict low-cost UAV solutions. In addition to the above, many of the existing works solely emphasize the perception of targets. For instance, Wang et al. [13] mainly applied a camera with gimbal and further merge GIS information for object detection and tracking.

Given the above, in general, most object tracking approaches on UAVs that rely on the traditional image post-processing techniques are not competent enough for real-time surveillance applications. Furthermore, UAVs are constrained with size, weight, and power (SWaP) limitations, and therefore in many of the state-of-the-art UAV technologies a single camera is usually deemed to be the relatively optimal sensor. Moreover, the notable advancement of computer vision technologies has enabled a prosperous area of research for the deep learning-based UAV system in contributing to the surveillance works. Lastly, with the limited field of view (FoV) of camera, it is also considered that the UAV should be capable of maneuvering so that a dynamic target is tracked and being contained within the FoV during surveillance missions. Therefore, we are motivated to develop a deep learning-based UAV system that accomplishes real-time and dynamic object tracking to achieving autonomous surveillance. Without the prior information of the environment, the proposed UAV system could use deep learning-based perception and filter-based 3D object pose tracking methods to monitor the activity of target objects in the surrounding environment during flights. In particular, the main contribution of this work is an autonomous object tracking UAV system for surveillance application, in which,

- real-time, learning-based object detection algorithm is integrated with the UAV embedded system to autonomous locate the desired object without human interference;
- a 3D pose tracking algorithm with object detection, stereo reconstruction techniques, and Kalman filter is implemented in a low-cost UAV system to recognize, locate and track the target object autonomously; whilst an UAV path planning is included for surveillance mission, which obeys the dynamic constraints for UAV to track and follow the target object movement;
- system experiments include both dynamic object and dynamic sensor, and the results validated good performance of the proposed system.

The following content of the paper is organized as follows: Section 2 introduces the relevant literature. Section 3 describes the overall hardware and software architecture of the UAV system. Section 4 and 5 explain the detailed methodologies of perception, tracking and maneuver. Section 6 presents and analyses the experimental results. The video footage of experiments and implementation codes are attached in the supplementary material.

## 2. Related Work

### 2.1. Object Detection

Object detection includes object localization and classification. Researchers have been attempting numerous approaches to achieving object detection over the last decades. Wang and Liu [14] described the traditional object detector processed pipeline of 4 stages, including (1) multi-scale sliding window, (2) hand-crafted features extraction, (3) classification by support vector machine (SVM) or AdaBoost classifier, and (4) non-maximum suppression (NMS), and combined bounding box to optimize object detection performance. However, traditional approaches encountered limitations such as low robustness and high inaccuracy for various geometric changes while also spending excessive computation costs for real-time operation. Other discrete object detection algorithms like point target detection and generalized contour search algorithms [15] showed better performance than the preceding approach. However, they still suffered limitations of accuracy, speed, cost, and complexity.

Deep learning-based approaches have emerged as the key breakthrough for object detection in computer vision and the UAV industries. The state-of-the-art object detection algorithms, particularly the convolutional neural networks (CNNs) series and ‘you-only-look-once’ (YOLO) series [16], are both derived from the DNNs. Andriluka et al. [17], Bejiga et al. [18] and Lygouras et al. [19] fused the CNN-based algorithms with onboard visual sensors of UAV to achieve real-time object detection in conducting search and rescue (SAR) missions. Meanwhile, Tijtgat et al. [20], Kyrkou et al. [21], and Feng et al. [22] employed ‘YOLO’ series algorithm as the object detection framework for real-time UAV applications. Deep learning-based approaches, both CNNs and ‘YOLO’ method, are deemed to be the powerful and prevailing object detectors embedded in the vision-based UAV navigation system.

Specifically, ‘YOLO’ took advantage of CNNs based architecture and applied single CNN on the whole image, generating bounding boxes coordinates, confidence level, class probability in one evaluation. Nevertheless, despite the accomplishment of real-time detection speed, ‘YOLO’ causes inaccuracy in object localization, especially for small or adjacent objects in images. Therefore, several updated versions of the ‘YOLO’ framework like YOLOv2 [23], YOLOv3 [24], YOLOv4 [25] were developed to improve speed, accuracy, and availability for embedded computing devices with limited computational resources.

The ‘YOLO’ object detection systems have high computational requirements, among which the powerful graphics processing unit (GPU) is a fundamental component. The computational resource on a UAV’s GPU remains the most difficult issue that causes slow speed and constrains the usage of state-of-the-art object detectors. Hence, Shafiee et al. [26] proposed a ‘Fast YOLO’ framework to speed up the object detection by 3.3 times. Additionally, Huang et al. [27] recommended the ‘YOLO-LITE’ that works well with non-GPU computers. Lastly, ‘YOLO v2-Tiny’ and ‘YOLO v3-Tiny’ by Redmon [23,24] and ‘YOLO v4-Tiny’ by Bochkovskiy [25] significantly reduce the network complexity of the original ‘YOLO’ framework. ‘YOLO v3-Tiny’ [24] delivers a higher attainable frame per second (FPS) and lower network size than the ‘YOLO’ model.

After an intensive investigation of the state-of-the-art computer vision research, this project utilized the latest ‘YOLOv4-Tiny’ on the UAV’s resource-limited onboard computer. Due to the SWaP constraints, we consider the high detection speed (high FPS) with acceptable precision and portability of C-based release to be an adequate and suitable solution to the proposed objectives. The detailed implementation of ‘YOLO v4-Tiny’ as the perception solution is elaborated in Section 4.

### 2.2. Object Tracking

Beyond object detection, the remarkable development of computer vision technologies in recent years encourages an exciting new array for object tracking applications. In addition to localizing and classifying the target object, object tracking involves the motion estimation or trajectory prediction of objects across a sequence of frames [28,29,30]. Nevertheless, it is deemed more challenging than object detection as it faces uncertainties and complexities in aspects including scene illumination changes, the abrupt motion of objects, occlusions, noise corruption in images, camera motion blur problem, and so forth [28,29,30]. Lee et al. [28] further pointed out that occlusion is the most common issue that happens in object tracking, regardless of its type (e.g., partial occlusion, full occlusion, inter-object occlusion), which leads to defects of tracking loss and identity switches. Occlusion means the tracked object is not available for camera to keep monitoring its motion state while the object is still present at the same scene. Lee et al. [28] also recommended fusion methods with linear or non-linear dynamics models to handle the occlusion problem.

In recent years, some researchers initiated ‘tracking-by-detection’ algorithm [31], in which the generative, discriminative, and hybrid statistical modeling were fused to improve the performance of object tracking. Nowadays, benefiting from the revolutionary enhancement of deep-learning, different learning-based tracking frameworks were presented, such as unsupervised deep-learning algorithm, pre-training network combined with correlation filter, siamese-based tracking network as well as the spatially supervised recurrent convolutional neural networks with YOLO and long short-term memory (LSTM) [32,33,34]. Another noteworthy development was the ‘DeepSort’ tracker proposed by Wojke et al. [35]. Specifically, ‘DeepSort’ applied CNN to the SORT (simple online and real-time tracking) framework that implemented Kalman filtering in image space and the Hungarian method. It learned the features of tracked object and predicted the future associated trajectories and positions of the objects of interest. The recent work from Punn et al. [36] demonstrated the positive results of using YOLOv3 with DeepSort tracking scheme to observe the social distancing situation. Both Kalman filter and the Hungarian method helped motion prediction and association of object tracking.

### 2.3. Unmanned Aerial Vehicle (UAV) Applications with Target Monitoring

Object tracking in image planes of camera and object tracking in vision assisted UAV systems are two different study fields as the latter requires additional relative control and coordination of UAV in flight [37]. The fast movement of airborne UAV, limited field of view (FoV) of onboard camera, and planning of UAV to maintain visible distance with goal objects are all essential considerations in planning object tracking by UAVs. In addition, the limited computational resource also creates the difficulties of object tracking on embedded systems. Ryan and Hedrick [38] used UAVs installed with infrared cameras to track the helicopter during SAR missions. The Kalman filter estimation was proven as an effective solution to predict a helicopter’s position and velocity. Rathinam et al. [39] applied the vision-based following systems on UAVs to autonomously track the path of river or coast; but they occasionally struggled with high error rate and low robustness. The better approaches in this field were the image feature processing with Kalman filtering [40] and the appearance-based tracking algorithm on color and depth data [41]. In addition to the above, Xu et al. [42] made a good paradigm in employing YOLO and JPDA on small-scale UAVs to achieve real-time multiple object tracking. On the other hand, some of the proposed systems, such as works carried out by Jayaweera and Hanoun [43], Han et al. [44] or Haugen and Imsland [45], focused more on the planning and control of the UAV system during a target-tracking mission, in which path planning or trajectory optimization tasks were carried out and addressed. However, from their literature, limited emphasis on the real-time perception methodology was given.

To summarize, deep learning-based object detector with filter-based method is considered as a novel and promising approach with high flexibility in categories of target objects, reduced occlusions, and real-time processing speed. As for this work, we utilized the YOLOv4-Tiny object detector and Kalman filter to perform target object tracking, and further proposed an efficient UAV planning to achieve a real-time accurate tracking and autonomous surveillance UAV system.

## 3. System Architecture

**Figure 1:**
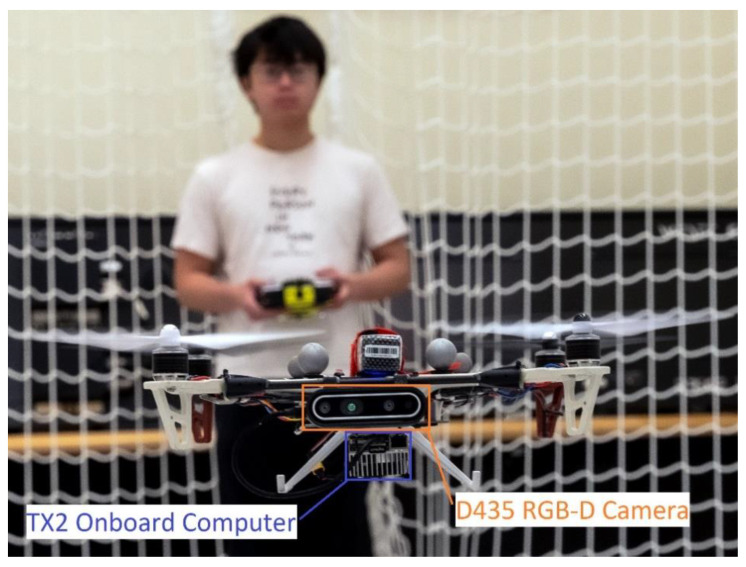
Prototype of proposed autonomous object tracking system.

To perform a surveillance mission, a camera that acts as optical sensor is essential. Hence, the main component of the vision-based system is an Intel RealSense D435 stereo camera for visual sensing and depth acquisition as it is proven to have light weight, wide FoV with a global shutter for moving camera motion, and high depth accuracy and stability. Besides, we employed a powerful GPU for embedded systems, the Jetson TX2 onboard computer to process deep learning-based algorithms on small-scale quadrotor UAV platform. The deployed flight controller is a Pixhawk 4 and an external VICON Mocap system is utilized for indoor visual localization. Moreover, the framework is supported by the robot operation system (ROS), using the *MAVROS* package to communicate PX4 flight controller and planner node at the onboard computer. Figure 1 shows the prototype of the proposed system.

**Figure 2:**
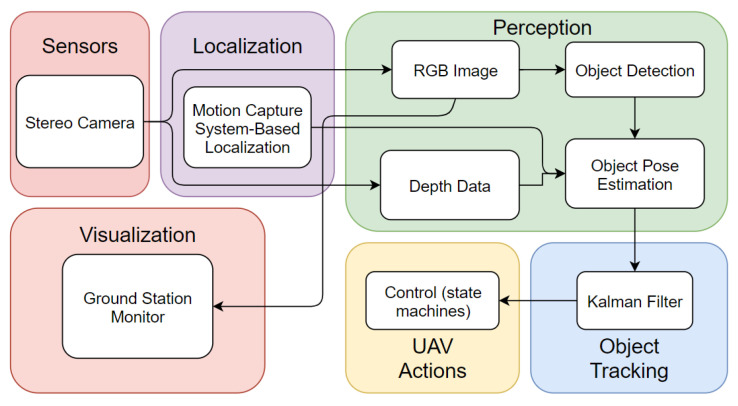
The software architecture of autonomous object tracking system.

The predominant software architecture of the designed autonomous object tracking UAV system consists of (1) perception module, (2) object tracking algorithm, (3) UAV maneuver, and (4) ground station visualization. In brief, the UAV system perceives the RGB image and the depth data, and with the deep-learning based object detector YOLOv4-Tiny, the drone can then recognize objects in its FoV. The generated 2D bounding boxes are fused with the depth measurement from camera and consecutive regions of interest (ROI) to obtain the 3D pose estimation of objects. A Kalman filter prediction module is integrated to help anticipating the motion of the tracked object. Lastly, a path planning component is incorporated with a finite state machine (FSM) to perform target tracking and following, while the preliminary visualization user interface is included. Figure 2 shows the overall software architecture.

## 4. Object 3D State Estimation

The foremost and essential procedure to perform object tracking would be precepting an object in a 3D world. We adopt a learning-based detector to generate 2D information and conduct 3D stereo reconstruction techniques. In this section, we will first discuss the adopted object detection method and further elucidate the 3D position information acquisition.

### 4.1. Object Detection

The UAV must be capable of identifying the target object independently. To achieve this, we employed the state-of-the-art YOLOv4-Tiny algorithm as our object detection solution due to the robustness, detection speed, and computational cost requirements during the tracking process. This subsection mainly discusses implementing YOLOv4-Tiny in the proposed UAV system, particularly the model training with the dataset and the 2D bounding box prediction.

#### 4.1.1. Dataset Establishment and Training

The first step in using the open-source ‘YOLOv4-Tiny’ would be the preparation of a customized dataset for training. Generally, each training class should have at least 2000 images. Meanwhile, to avoid overfitting and improve training results, it is suggested to have a validation dataset to provide an unbiased assessment of a model fit on the training dataset. Hence, the entire dataset would comprise subsets of the training set and the validation set.

**Figure 3:**
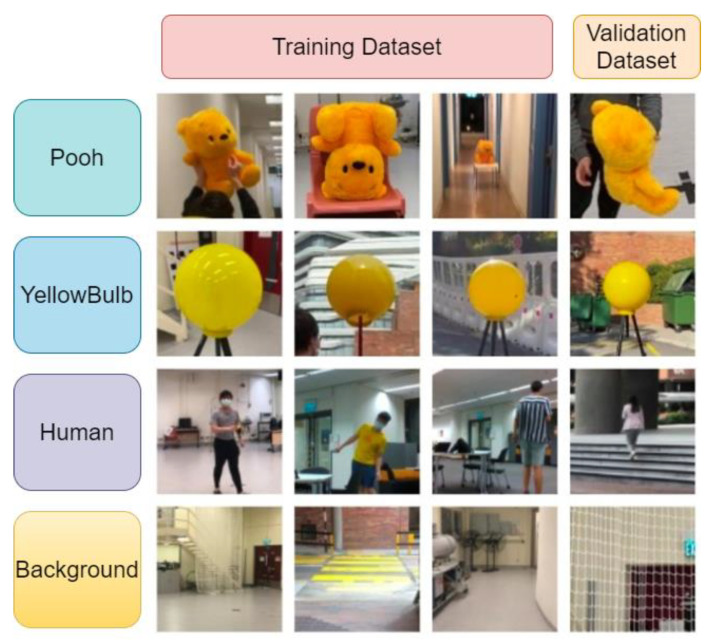
Random image samples from the custom dataset.

Our model aims to detect three classes of objects (i.e., Winnie-the-Pooh soft toy, yellow bulb ball and human). The human class usually appears as the target object in a surveillance mission, while the other two classes are included for experimental convenience. To improve the detection performance of the trained model, the custom dataset should contain images with random and dissimilar illumination conditions, scales, view of angles, aspect ratios, resolutions, and backgrounds. Additionally, it is also considered that the system could be integrated with other custom-trained models for specific surveillance missions. We established a dataset including 13,500 images, composed of 2000 training images plus 500 validation images (4:1 ratio) for each class, as well as 6000 background images with no target object. The 6000 background pictures are designed as negative images to raise the model’s accuracy because it will learn to detect no object in a scene, thus reducing false positive (FP) results. Additionally, within the training dataset, many images contain multiple objects (i.e., Pooh, yellow bulb ball, human) in a single frame, which would enhance the detection accuracy in the scenario that multiple target objects appear in the same scene. Some representative images of our dataset are shown in Figure 3.

Data labelling is an important process indicating interest in the object and providing the ground truth bounding box of the image dataset, so that the IOU (intersection over union) and the confidence score can be calculated, and the optimal weights for the model can be developed. We manually labelled the bounding boxes and the corresponding class names (i.e., Pooh, yellow bulb, human) on all 13,500 images by employing an annotation tool called LabelImg designed by Tzutalin [46].

During the training process, the discrepancy is calculated by loss function, and the result is referred to as loss or cost through the continual comparison on a large number of iterations. Two important metrics that quantitatively measure the performance in the training process are the mean average precision (mAP) and the loss. In short, the intentions of the training are to maximize the mAP and to minimize the loss. Empirically, a training process is deemed as effective if the mAP reaches acceptable value and levels off after a certain number of training epochs. As the iteration number of the model increases, the mAP also gradually increases as the model is more capable of detecting the target object accurately. We observed the changes of loss throughout the process, as the model executed an optimization of sum-squared error loss and multi-part loss function to reduce the overall loss. We trained the model until there was no significant drop of loss, which indicated that the discrepancies between the model predictions and the ground truths were sufficiently low.

#### 4.1.2. Two-Dimensional (2D) Bounding Box Prediction

In real-time object detection, YOLO predicts the 2D location of a detected object by generating 2D bounding boxes on every single frame of the streaming video input. Since the upgrade of YOLOv2, the k-means clustering method and anchor-box mechanism were adopted in predicting 2D bounding boxes on objects. Using the anchor box to predict bounding box could increase the average IOU for each grid cell and thus enhance the overall accuracy of object localization.

There is a pre-defined number and shape of anchor boxes on each grid cell through the k-means dimension clusters method. For instance, if the default number is 3, the YOLOv4-Tiny outputs 6 × 6 × 3 anchor boxes on a 6 × 6 feature map. The center of the anchor box is always located at the center of its respective cell. The shape is normally rectangular in different orientations and aspect ratios. Every anchor box predicts class and “objectness”. Among all numbers of anchor boxes on different grid cells, only the anchor boxes which are predicted to contain the object (i.e., objectness = 1 with a certain confidence score) would be kept. Then, only the anchor boxes that have the highest similarity and closest shape to the ground-truth box of a target object would be kept as positive anchor boxes for further processing. In other words, the selection of the anchor box depends on the confidence score output of the network and the following non-max suppression (NMS) technique, or more explicitly, the highest IOU between the ground-truth box and the selected anchor box. After acquiring the anchor boxes for a particular object, the anchor boxes with score values higher than the set confidence threshold values are further transformed to the final predicted bounding box using a parameter regression function.

According to Redmon and Farhadi [33], YOLO adopted the following computation in transforming the anchor box to the predicted bounding box. One anchor box generates one bounding box with four parameters:

(1) $$b_{x}=\sigma \left(t_{x}\right)+c_{x}\;$$

(2) $$b_{y}=\sigma \left(t_{y}\right)+c_{y}$$

(3) $$b_{w}=p_{w}^{e_{t_{w}}}$$

(4) $$b_{h}=p_{h}^{e_{t_{h}}}$$

- $t_{x\;},t_{y}\;,\;t_{w}\;,\;t_{h}$ are coordinates of predicted bounding box in terms of x position, y position, width, and height, which are not finalized bounding box coordinates.
- $c_{x\;},\;c_{y}\;$are the offset of cell from the top left corner of the image.
- $p_{w}$ is the width and $p_{h}$ is the height of the predicted prior anchor box.
- *σ* is the sigmoid function applied to constrain the offset range between 0 and 1.
- $b_{x}\;,\;b_{y}\;,\;b_{w}\;,\;b_{h}\;$are the finalized parameters of bounding box, where $b_{x}\;$and $b_{y}\;$are the center coordinates, $b_{w}$ and $b_{h}\;$are the width and height respectively.

**Figure 4:**
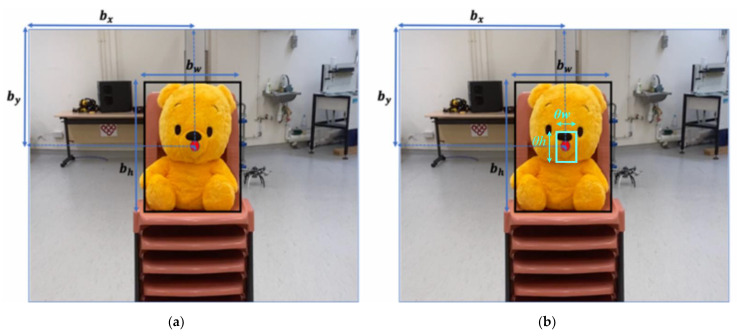
(**a**) Bounding box coordinates on the image plane produced by YOLOv4-Tiny. (**b**) The region of interest for depth information $S_{i}$.

Figure 4a shows an instance of prediction result generated by YOLOv4-Tiny.

### 4.2. Three-Dimensional (3D) Pose Estimation

From Section 4.1, we save the predicted bounding box as $S_{ROI}$. We then recover the 3D pose of the object to conduct dynamic tracking by the following information: (1) the coordinates of the object on 2D frame, (2) the depth information retrieved from the stereo camera. We first generate an inner rectangle $S_{i}$ by shrinking $S_{ROI}$ with scaling factor θ:

(5) $$S_{ROI}=\left[c_{x}\;c_{y}\;w\;h\right],\;$$

(6) $$S_{i}=\left[c_{x}\;c_{y}\;\theta w\;\theta h\right].$$

The acquired $S_{i}$, as shown in Figure 4b, will then play as the region of interest (ROI) on depth for depth information acquisition. From the depth image acquired by the stereo camera, we first filtered out the unfilled pixels and averaged the remaining depth data in $S_{i}$. We then assumed the averaged depth value s as the distance between the observer and the target object. Subsequently, with the bounding boxes coordination, we conducted coordination transformation and obtained the relative pose from the camera and the global pose in the world frame. The frame transformation equations are as follows:

(7) $$s{\left[u\;v\right]}^{T}=K\cdot \left[\begin{array}{c} X_{i}^{C}\\ 1 \end{array}\right],$$

(8) $$\left[\begin{array}{c} X_{i}^{W}\\ 1 \end{array}\right]=T_{B}^{W}T_{C}^{B}\left[\begin{array}{c} X_{i}^{C}\\ 1 \end{array}\right],T_{B}^{W}T_{C}^{B}\in SO\left(3\right)\;,$$

where *u* and *v* are the pixel coordination of the $S_{i}$, *K* is the intrinsic camera matrix, $X_{i}^{C}$ is the object pose vector in camera frame, while $X_{i}^{W}$ being the object pose vector in the world frame. In particular, the transformation matrices are:

(9) $$T_{C}^{B}=\left[\begin{array}{cccc} 0 & 0 & 1 & 0\\ -1 & 0 & 0 & 0\\ 0 & -1 & 0 & 0\\ 0 & 0 & 0 & 1 \end{array}\right]$$

(10) $$T_{B}^{W}=\left[\begin{array}{cc} \begin{array}{cc} r_{11} & r_{12}\\ r_{21} & r_{22} \end{array} & \begin{array}{cc} r_{13} & o_{x}\\ r_{23} & o_{y} \end{array}\\ \begin{array}{cc} r_{31} & r_{32}\\ 0 & 0 \end{array} & \begin{array}{cc} r_{33} & o_{z}\\ 0 & 1 \end{array} \end{array}\right],$$

in which $r_{ij}$ is the element in the rotation matrix of the attitude of the observer, and $o_{x},\;o_{y}\;,\;o_{z}$ are the current position of observer (UAV) with respect to the world frame. The rotation of a coordinate frame is usually expressed in either rotation matrix or quaternion representation.

## 5. Filter Based Tracking and UAV Maneuvers

### 5.1. Relative Pose Estimation

We utilized the YOLOv4-Tiny framework as it possesses a good trade-off between speed and accuracy. Nevertheless, the higher FPS also indicates that the accuracy has been, to some extent, yielded. Furthermore, as both the states of the target object and the quadrotor are dynamic, the pose estimation based on Section 4 is considered insufficiently robust. In a surveillance mission, it is not guaranteed that the target object could always be captured in the FoV, as there might be false positive or false negative results; and occasionally, partial or full occlusion might also occur. In particular, although severe occlusions might not be resolved with such a method, it is deemed that the proposed method would suffice to deal with occlusions that occurred within a short duration of time. To address the above issues, we utilized the Kalman filter to increase the tracking performance.

#### 5.1.1. Kalman Filter

As the Kalman filter is frequently substantiated to be a sufficiently robust solution in the robotics field, it is chosen to be a critical module in the proposed system.

We first established the state vector of the object with the relative positions and velocities from the camera, i.e., the x, y, and z coordinates in the camera coordination frame. The state-space vector is shown as:

(11) $$x_{k}={\left[p_{k},\;u_{k}\right]}^{T},$$

where $x\left(k\right)\in R^{6}$ and T represents the matrix transpose. We further considered that the target’s dynamic state varied with nearly constant velocity (NCV), and assumed that all the states, measurements, and noises followed the Gaussian distribution. Therefore, we could then describe the object’s dynamic system in the form of Kalman filter. The following content shows the discrete linear equation of the target object, and the measurement expression:

(12) $$x_{k}=A\left(\Delta t\right)x_{k-1}+w_{k}$$

(13) $$z_{k}=Hx_{k}+v_{k}\;,$$

in which A(Δt) is the transition matrix, $w_{k}$ is the process noise, $z_{k}$ is the measurement from the detection module, *H* is the measurement matrix, and $v_{k}$ is the measurement noise. The system can then be further divided into two steps: time update (prediction) and measurement update (correction).

Time update (prediction):

(14) $${\hat{x}}_{k}^{-}=A{\hat{x}}_{k-1}+Bu_{k-1}$$

(15) $$P_{k}^{-}=AP_{K-1}A^{T}+Q.$$

Measurement update (correction):

(16) $$K_{k}=P_{k}^{-}H^{T}{\left(HP_{k}^{-}H^{T}+R\right)}^{-1}$$

(17) $${\hat{x}}_{k}={\hat{x}}_{k}^{-}+K_{k}\left(z_{k}-H{\hat{x}}_{k}^{-}\right)$$

(18) $$P_{k}=\left(1-K_{k}H\right)P_{k}^{-}.$$

Specifically,

(19) $$Q=E\left[w_{k}\;w_{k}^{T}\right]$$

(20) $$R=E\left[v_{k}\;v_{k}^{T}\right].$$

The two matrices (*Q* and *R*) are the covariance matrices of noises ($w_{k}$ and $v_{k})$, and $P_{k}$ is the error covariance matrix.

The Kalman filter mainly resolves the problem of the estimation of states; from the above equations, the objective is to obtain the filtered result ${\hat{x}}_{k}$ at every discrete time step k. Mostly, the filter makes educated estimations based upon the following: (1) the predictions (${\hat{x}}_{k}^{-}$) from previous states, (2) the measurement ($z_{k}$) at each frame (elucidated in Section 4), and (3) the optimal Kalman gain ($K_{k}$). The process is iterative, and its performance has been empirically determined to be satisfactory in the designed surveillance UAV system, whose results will be presented in Section 6.

#### 5.1.2. Overall 3D Tracking Algorithm

To track the target object, the system will execute the following working pipeline. After the video stream frame is retrieved, the deep learning model will generate bounding boxes with every object having a corresponding confidence score. Nevertheless, only the bounding box with the target object class will be tracked. If multiple objects are found, the designed software will only consider the bounding box with the largest ROI area and discard the rest. Due to the relatively lower mAP of YOLOv4-Tiny, to avoid false positive detection the system will only take the bounding boxes with a confidence score higher than 0.75 as direct information output. Those outputs with a confidence score lower than 0.75 will be fed to the update equation of the Kalman filter correction step shown in Equation (17). The system will then take the posterior estimates as the final output. The threshold of 0.75 is empirically determined. Nevertheless, in some scenarios, temporary object occlusion or false negative detections could happen, and the system might lose track of the object. In such a situation, the Algorithm 1 will take the prior results from the prediction step and deem it as the perception result. The following pseudo-code shows the overall 3D tracking algorithm:
**Algorithm 1: 3D Yolo-KF-Tracking****Notation:** object states $x_{k}$, measurement $z_{k}$, Kalman filter KF, image set F
**Input**: image F
**while**
*true*
**do**
  *Object-Detection* (F)
  **if** object detected **then**
    trigger and initiate KF 
    **break**
  **else**
    **continue**
  **end if**
**end while**
**while**
*true*
**do**
  KF.predict()
  *Object-Detection* (F)
  **if** object detected **then**
    **if** confidence score > 0.75 **then**
      ${\hat{x}}_{k}$ = $z_{k}$
      KF.update ($z_{k}$)
    **else**
      KF.update ($z_{k}$)
      ${\hat{x}}_{k}$ = KF.update ($z_{k}$)
    **end if**

  **else**
    ${\hat{x}}_{k}$ = KF.update (KF.predict())
  **end if**
  **Output**: ${\hat{x}}_{k}$ (posteriori estimate)
  **continue**
**end while**

### 5.2. Finite State Machine Definition

In a surveillance mission, to capture the target object within the FoV, the UAV computes the relative position of the target object from its FoV and determine the reactional maneuver. In particular, all the relative positions are in the UAV’s camera coordination system. By determining the moving trend of the target object on each of the three axes, the UAV would define its states, formulating a finite state machine (FSM). Specifically, the system is designed to have two parallel state machines: one for resolving the attitude and altitude of the camera FoV, and the other for modifying the relative distance between UAV and the target. In addition, we have designed a position controller for the planning model of the proposed system, where the UAV will be following a series of discrete waypoints generated by the state machine.

The following items are the state definitions and the corresponding UAV maneuver based on the sequence of the states in a surveillance mission.

**Initialization:** starting from ground, the camera is turned on when the whole system is being initialized. The UAV will then take off to a certain altitude and start to search for the target object.

**Sway and Search:** after going airborne, the UAV will then sway for 360 degrees to search for the object. To avoid a severe motion blur that affects the perception performance, the angular velocity of the swaying is set to be conservatively low, which is constrained to be lower than $V_{\theta_{max}}$.

**Track and Hover:** after locating and locking the target, the UAV will enter “track and hover” mode. During this stage, the system will be based on the consecutive frames from the camera input and determine whether the target is dynamic or not. If the target is observed as “static”, the surveillance UAV will continue to hover.

**Track and Sway:** for a surveillance assignment, we consider that the center axis of the camera should be aligned with the target. By doing so, the system can prevent the target from exiting the FoV in a short duration of time. Therefore, when being in the state of “track and hover”, if the target is observed as “horizontally dynamic”, the UAV will try to sway around, keeping the target object staying within the vicinity of the center. However, in order not to exceed the dynamic feasibility of the UAV, the angular velocity has also been restricted to be less than $V_{\theta_{max}}$.

**Track and Climb or Descend:** similar to the above, the UAV will decide to climb or descend, depending on the relative position to the target object. The vertical velocity is limited within $V_{z_{max}}$ to maneuver within the dynamic constraints.

**Track and Forward or Backward:** to guarantee a collision-free flight, the UAV should maintain a certain safety distance $R_{safe}$ with the target object. Nevertheless, in order not to lose the object, it is deemed that the UAV should be within a surveillance radius $R_{sur}$. Therefore, based on the inputs from the stereo camera, the system will calculate the depth data and determine whether the gap between them lies in the scope of $R_{safe}\;$and $R_{sur}$ and further decide the reactional movement. The moving velocity, analogously, should not exceed $V_{x_{max}}$.

The “x” and “z” in the subscript indicate the X and Z axis in the body frame. The system could function simultaneously in one or more states. For instance, if the target object is moving further, whilst travelling leftwards from the camera view, the system will be in both “Track and Sway” and “Track and Forward”.

**Lost and Await:** it is not guaranteed that the object could always be tracked. Therefore, we have designed a fail-safe mechanism. If the object is lost for too many frames, the UAV will enter the mode “lost and await” and hover until the object returns to FoV, or land after the waiting time exceeds the threshold.

**Land:** the UAV will land after the target object is lost for too many frames. It will try to return to its home position and land.

## 6. Experiment Results and Discussions

To validate the proposed UAV system, we conducted experiments through a strategy of gradual phases. Before the fast development of deep learning, object tracking has usually been separately discussed from object detection. Nonetheless, with the rise of robust detectors, researchers have increasingly deployed the “tracking by detection” method, and this has led to a convergence of difference between object detection and object tracking fields. Therefore, conventionally, for recent object tracking works, only tracking performance will be discussed. Nevertheless, as this work is based upon a self-generated dataset, we assert that it is necessary to discuss the training result of our detection model.

In this section, we first assessed the performance of the trained model on a Jetson TX2 onboard computer, where the YOLOv4-Tiny model was trained via the Darknet open-source framework through Google Colaboratory. As mentioned, we then observed the robustness of the proposed tracking algorithm on a 2D streaming video by exploiting several quantitative analysis techniques for object tracking. Lastly, we carried out intensive flight tests on a self-assembled quadrotor platform and evaluated the overall performance.

### 6.1. Training Result of YOLOv4-Tiny

The surveillance task starts with object detection. We employed the YOLOv4-Tiny model to perform object detection. The YOLOv4-Tiny model will output a prediction bounding box which classifies the detected object into a certain category and indicates the location of that object. The goal of the experiments is to validate the object detection performance using our trained model, which is critical for subsequent UAV pose estimation. The quality of using a ‘YOLO’ framework in operating real-time object detection as well as 3D pose estimation significantly depends on the training result of the YOLOv4-Tiny model on a custom dataset. Two factors, detection speed and accuracy, play dominant roles in judging the model training result. The model training process lasted for 6000 iterations at which the training loss did not decline any further. Since different neural network resolutions could influence the model precision, we trained our YOLOv4-Tiny model with different resolutions (i.e., 320 ×320, 416×416, 512×512, 608×608) to evaluate the best model performance. The comparison of the four input resolutions in terms of accuracy and detection speed is demonstrated in Table 1. Meanwhile, since the UAV surveillance task relies on real-time perception solutions to address object detection and tracking problems, the detection speed and accuracy need to be balanced such that the UAV can consistently detect and track the object with negligible delay and sufficient accuracy. Thus, a comparison between YOLOv4-Tiny and YOYLOv4 models of the same network resolution was made to examine accuracy and speed. Table 1 summarizes the training results.

Notably, larger input resolutions will increase the best possible mAP but will inevitably slow down the training process and the detection speed. Thus, it is not necessary to train higher input resolution as we achieved acceptable speed and accuracy at 608×608, at which the mAP is 80.20% with intersection of union threshold of 0.50 (AP_50_). However, its FPS is slightly lower than that of resolution 512×512. Furthermore, when comparing YOLOv4-Tiny to YOLOv4 model, we conclude that the YOLOv4-Tiny model generates a moderately lower mAP but much higher FPS. Since the object detection speed (FPS) should be of more importance in real-time autopilot operation, we therefore chose the YOLOv4-Tiny model with the resolution 512×512 as a good balance between detection accuracy and speed.

In Section 4.1.1, it was asserted that 6000 negative images play a crucial role in the process of custom model training. Therefore, a separated model of YOLOv4-Tiny was trained in order to validate the statement. From Table 2, it could be observed that mAP without the background pictures (negative images) turned out to be lower in all sizes of models, from 320 × 320 to 608 × 608.

Once the training process was completed, we assessed the model performance of detecting target objects on real-time videos captured on Intel RealSense D435i stereo camera. The trained model was robust under various environments and low false positives and low false negatives were found in the detection results. After assuring the validity of our trained model, the object tracking was then successively assessed.

### 6.2. Tracking Performance on Target Object

Some of the most common ways to evaluate the tracking performance of an algorithm are precision plots and success plots [47]. Therefore, the center error between the ground truth and tracked targets as well as the IoU (intersection over union) values were measured and calculated. Nevertheless, it is deemed to be unsuitable if all benchmark algorithms are compared with this work, as: (1) the camera could be constantly moving and giving occasional severe motion blur, while most of the other proposed research were designed with a video stream with the FoV being fixed; and (2) the work focuses on a customizable surveillance UAV system, in which it is preferred to assess the system and its algorithm on an embedded computation unit (with a suitable real-time speed); however, many of the state-of-the-art methods require high computation power. Hence, we only compared our algorithm with Opromolla et al. [48] and Peixoto et al. [49], where they deployed similar tracking techniques based on the YOLO detector. The compared system was able to be executed on the designed hardware architecture in real time.

Robustness of the tracking module was validated the on 2D video stream, in which the custom object was fully captured in most frames. The videos mainly consisted of several pre-recorded clips retrieved manually on campus prior to our flight tests, with the camera’s ego-motion being both static and dynamic. It is deemed that the target object in the video has been sufficiently exposed to different environmental backgrounds, illumination conditions, and different capture angles as well as distances. The video frames input was 640 × 480 and a total of 2767 frames were collected. The ground truth was labelled manually during the image post-processing.

We first calculate the center location error (CLE), i.e., the Euclidian distance between ground truth and tracker, by the following equation, with R being the bounding boxes of ground truth and tracker, and X being the states of the bounding boxes:

(21) $$\Delta \;\left(R_{G},\;R_{T}\right)=\|X_{G}-X_{T}\|\;$$

**Figure 5:**
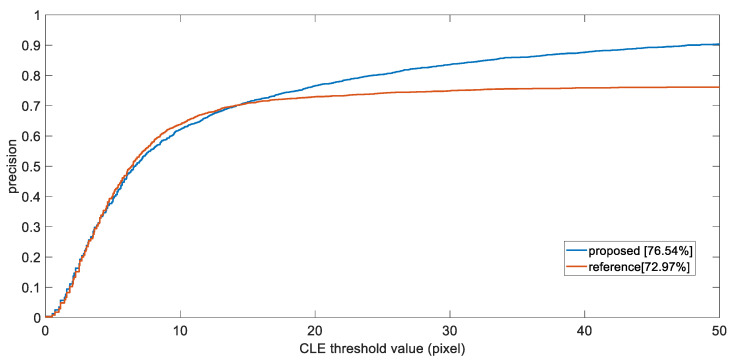
The precision plot of one-pass evaluation (OPE).

We then plot the precision plot of one-pass evaluation (OPE), where the *x*-axis is the center location error threshold, and y being the percentage of the frames whose center distance lies within the threshold. Additionally, we consider the precision score at threshold value 20 as the final representation of precision. Figure 5 shows the precision plot of OPE.

**Figure 6:**
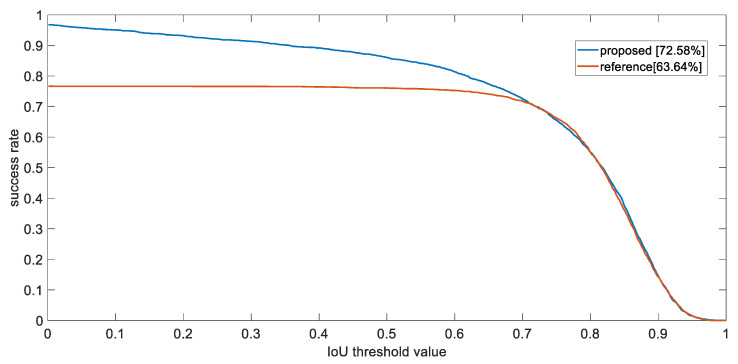
The success plot of the proposed tracking method and compared system.

As observed, the proposed method has outperformed the comparison set, as it achieved a precision rate of 76.54% at CLE threshold equals to 20, whereas the other being 72.97%. During the experiments, our method shows higher robustness for target object, as it managed to continuously follow the object for most of the time, even when object is occluded, or being captured with occurrence of motion blur. The success plot of OPE by calculating the IoU by

(22) $$S=\frac{\left|R_{G}\cap R_{T}\right|}{R_{G}\cup R_{T}}$$

is shown in Figure 6. Similar to precision plots, the *x*-axis of success plot is the threshold of IoU value, whilst the y axis being the percentage of frames that exceed this threshold. Figure 6 also indicates that our system has outperformed the other, as the area under curve (AUC), or average precision (AP) has been calculated to be higher than the other, being 72.58% and 63.63%, respectively.

As we retrieved the depth information based on the bounding boxes, both center location and region of interested (ROI) generated by the tracking algorithm matter. From above, the proposed tracking algorithm achieves an acceptable precision rate and average precision that guarantees a certain robustness.

Moreover, as the UAV surveillance system could be either hovering, swaying or producing linear motion, it is required that the performance difference between static and dynamic states should not be significantly high. Table 3 further compares the performance based on the root mean square error (RMSE) of the center location when the ego-motion of the camera is dynamically different.

It can be observed that although the RMSE values are apparently affected by the camera’s motion, as the RMSE values for trial 4, 5, and 6 are higher than the static trials. The value between the two is considered to be controllably near, as center location RMSE are all lesser than threshold = 20. Hence, we conclude that the overall discrepancies lie in an acceptable scope, making our system sufficiently robust under different state machines.

### 6.3. Flight Experiment in Indoor Environment Aided with External Locolization

The flight tests were conducted under a Vicon arena with the size of 6 m × 4.6 m. To simulate a surveillance mission, we have assigned the UAV to search, track, and follow the “Pooh” class object. During the experiment, we tried to move the target object around while the quadrotor maneuvered in order to track and follow the object. Both object and camera were constantly moving such that the difficulty of pose estimation was raised. In addition, we also included occlusion scenarios, as we intentionally trespassed the space between the UAV and the object. Furthermore, due to the cluttered environment, the parameters were conservatively set for safety reasons. Table 4 shows the values of the tuned parameters.

**Figure 7:**
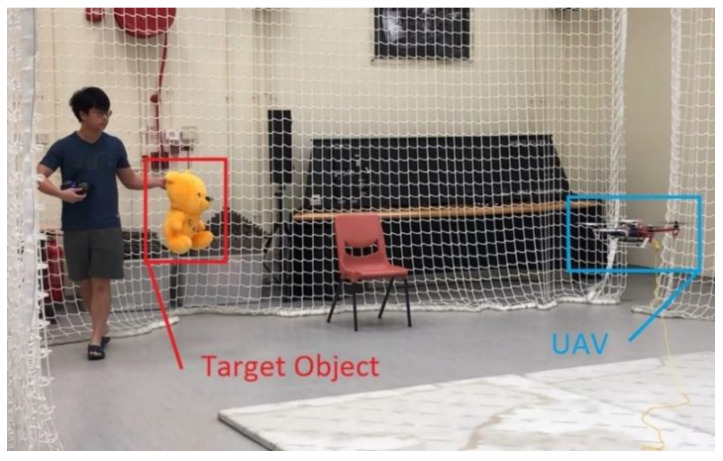
Flight test under the motion capture arena.

**Figure 8:**
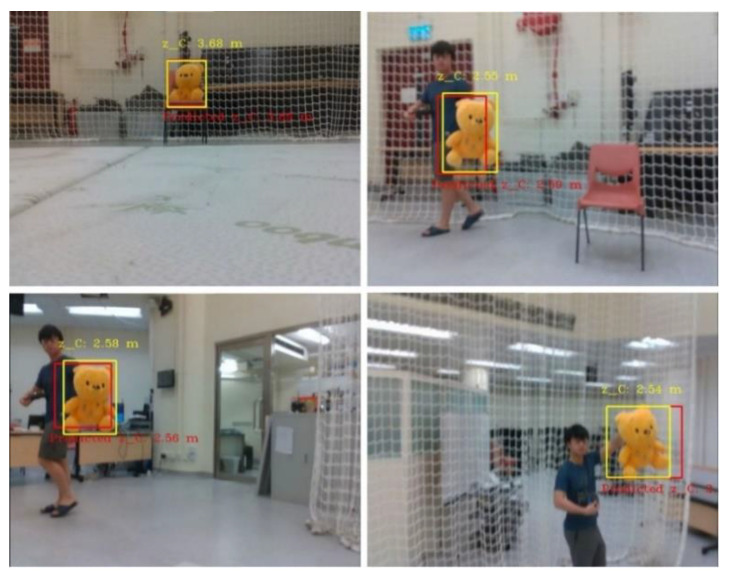
First-person views of the unmanned aerial vehicle (UAV) system during the tracking mission (the yellow bounding boxes are the detection generated by YOLO-Tiny, while the red being the predicted state of the object).

**Figure 9:**
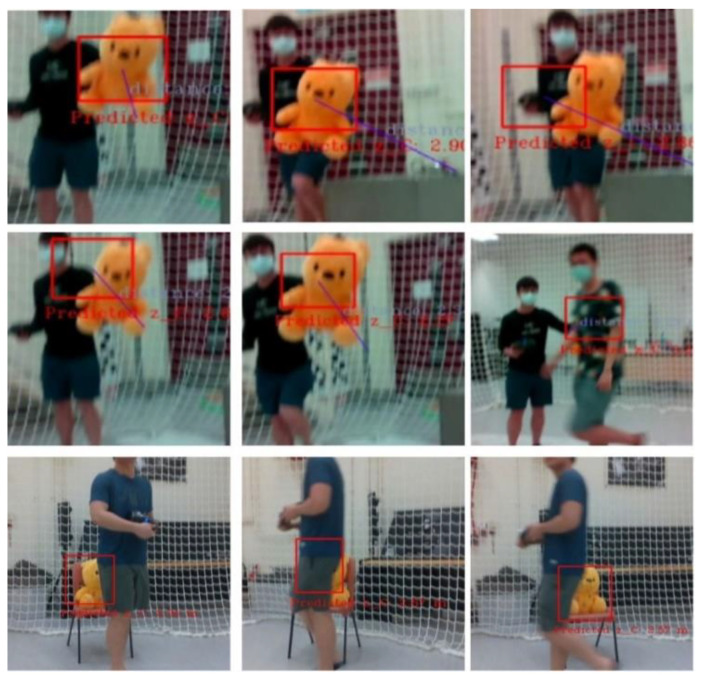
Frames where the target object is not detected or fully/partially occluded.

As this work focused on integrating a perception to reaction, end-to-end surveillance system, we first validated the overall flight behavior as shown in Figure 7, Figure 8 and Figure 9. During intensive trials, finite state machines were executed normally, even when the objects were occluded or not detected (false negative detections). The experiments are recorded and attached as the Supplementary Materials Video online.

**Figure 10:**
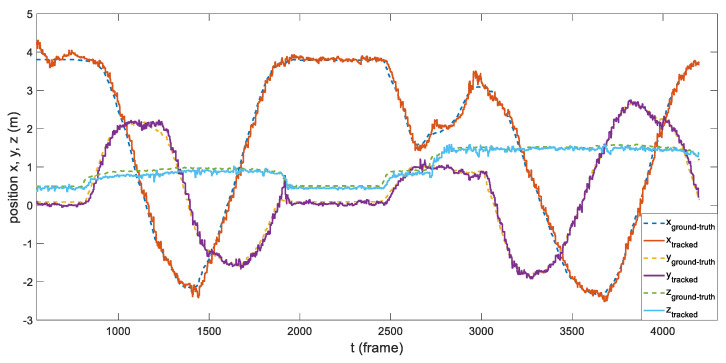
Comparison between the ground truth (retrieved by motion capture system) and the object position estimation.

**Figure 11:**
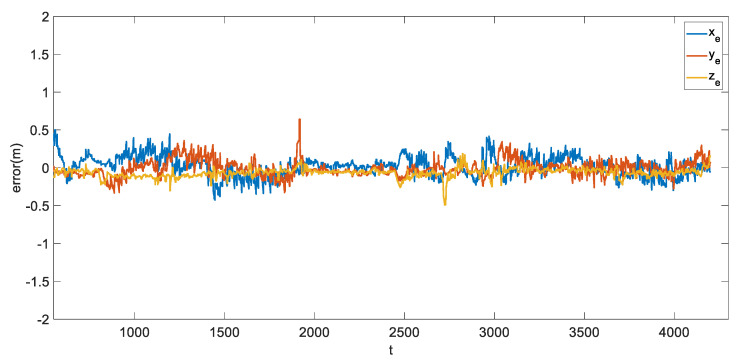
Error throughout the mission time.

To further evaluate the system, we compared the estimated dynamic position and the ground truth of the tracked object. As shown in Figure 10 and Figure 11, the system was able to track the object’s pose in the 3D space for most of the time. Despite having jittering and occasional drifts, the proposed tracking algorithm could still relocate the object after several frames.

In Figure 11, the error stays within 0.4 m in all axes of the world frame for most of the time. We further calculate the RMSE and MAE with results shown in Table 5.

Compared to other 3D object pose state-of-the-art estimation systems [22,50], which focused on static objects instead of dynamic, the proposed method possesses slightly higher error but is robust enough for real-time dynamic position estimation. Additionally, during a surveillance mission, as the distance between the UAV and the target object might not be consistent, accuracy discrepancies under different object distances were further analyzed. As shown in Table 6, it can be concluded that the performance proposed method does not significantly deteriorate when the object distance is increased.

**Figure 12:**
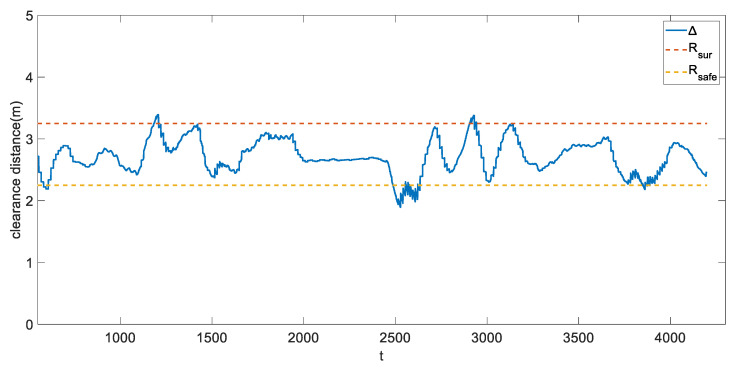
The clearance distance Δ (the relative gap between the UAV and dynamic target object) during the experiment.

To achieve a collision-free surveillance mission flight, we have defined the state machines in Section 5.2. For further evaluation, we then plotted the clearance distance throughout the flight. It is believed that the distance between the UAV and the target object should be maintained within $R_{safe}$ and $R_{sur}$, which are respectively 2.25 m and 3.25 m. Figure 12 shows the relative distance between the two.

As observed from Figure 11, most of the time the quadrotor stays within the scope of $R_{safe}$ and $R_{sur}$. Although the quadrotor may sometimes exceed the pre-defined boundaries during the flight test, it still successfully fell back after a while. We consider the sparse overshooting periods, which were caused by the abrupt movement of the target object, would not significantly affect the overall performance and conclude that the proposed method could achieve a real-time, maneuverable and autonomous UAV surveillance system.

## 7. Conclusions

In this work, we established an autonomous UAV tracking system for assisting surveillance using a deep learning-based approach. We employed the YOLOv4-Tiny to train a model for object detection based on our custom dataset. A Kalman filter was leveraged to aid the YOLOv4-Tiny for 3D pose estimation to increase the tracking performance. The Kalman filter was also responsible for tackling the common problems in aerial tracking, such as false positive or false negative detection and occasional occlusions. Additionally, an UAV maneuver state machine was incorporated to conclude the fully autonomous system. The proposed autonomous UAV system does not require prior knowledge regarding the external environment or target objects. System modules were evaluated through extensive experiments in both virtual and real environments. The experimental results have validated the system feasibility and robustness for object surveillance tracking.

For future work, the vision-based and GNSS-based positioning systems will be merged into the proposed UAV system. The aforementioned independent localization/positioning component allows the proposed system to carry our missions in both indoor and outdoor environments. In addition, it is also considered that more work on the path planning module could be extended, in which the trajectory should be optimized based upon the following: target motion prediction, dynamic and static obstacles constraints, as well as UAV robot physical limitations. Such work will secure the target object within the UAV’s camera FoV with a higher likelihood (occlusions or loss of target reduced), whilst performing a collision-free mission, augmenting the applicability of the UAV tracking system.

## Figures and Tables

**Table 1:** Performances of YOLOv4-Tiny and YOLOv4 with respect to different resolutions.

Method	Backbone	Size	mAP@0.50 (AP_50_)	FPS
YOLOv4-Tiny	CSPDarknet-53-tiny	320 × 320	74.85%	16.63
		416 × 416	77.21%	16.19
		512 × 512	79.36%	16.31
		608 × 608	80.20%	14.34
YOLOv4	CSPDarknet-53	416 × 416	97.09%	3.16

**Table 2:** Comparison between models trained with/without negative images.

Backbone	Size	mAP@0.50 (AP_50_) with Negative Images	mAP@0.50 (AP_50_) without Negative Images
CSPDarknet-53-tiny	320 × 320	74.85%	59.97%
	416 × 416	77.21%	62.87%
	512 × 512	79.36%	66.08%
	608 × 608	80.20%	66.40%

**Table 3:** Comparison of root mean square error (RMSE) between static and dynamic camera ego-motion.

Experiments	No. of Consecutive Frames	Camera Ego-Motion	RMSE (Pixels)
Trial 1	487	Static	14.30
Trial 2	356	Static	8.09
Trial 3	220	Static	9.37
Trial 4	705	Dynamic	17.88
Trial 5	466	Dynamic	16.21
Trial 6	533	Dynamic	18.47

**Table 4:** Defined parameters for flight test.

Parameters	Value
θ	0.15
$V_{\theta_{max}}$	0.2 rad/s
$V_{z_{max}}$	0.4 m/s
$V_{x_{max}}$	0.4 m/s
$R_{safe}$	2.25 m
$R_{sur}$	3.25 m

**Table 5:** Calculated RSME and mean absolute error (MAE) of the dynamic object position estimation.

Error Evaluation	X (m)	Y (m)	Z (m)
RMSE	0.1322 m	0.1072 m	0.0896 m
MAE	0.1033 m	0.0812 m	0.0728 m

**Table 6:** Calculated RSME and MAE of the dynamic object position estimation with object distances being different.

Object Distance	1–3 m			8–10 m		
Error Evaluation	X (m)	Y (m)	Z (m)	X (m)	Y (m)	Z (m)
RMSE	0.1322 m	0.1072 m	0.0896 m	0.1850 m	0.1286 m	0.1116 m
MAE	0.1033 m	0.0812 m	0.0728 m	0.1172 m	0.1019 m	0.1002 m

## Data Availability

Not applicable.

## Supplementary Materials

The following are available online at https://youtu.be/tY16YnZQoB4, https://youtu.be/vO2N5aY1nE4, Video: Dynamic Object Tracking on Autonomous UAV System: for Surveillance Applications, https://github.com/HKPolyU-UAV/AUTO (accessed on 29 October 2021), Source code.
